# Molecular Genetics of *Hepatozoon canis* With a Note on Effect of Parasite on the Complete Blood Count Profile of Canine Hosts

**DOI:** 10.1002/ece3.74039

**Published:** 2026-08-05

**Authors:** Madiha Rasool, Muhammad Latif, Ioannis A. Giantsisis, Muhammad Ali, Hira Muqaddas, Muhammad Naeem, Sami Ul Haq, Gehan M. Elossaily, Esmael M. Alyami, Mona Alsolami, Ousman Brahim Mahamat, Irfan Ullah, Asmat Ullah Khan, Adil Khan, Furhan Iqbal

**Affiliations:** ^1^ Institute of Zoology Bahauddin Zakariya University Multan Pakistan; ^2^ Department of Zoology The Women University Multan Multan Pakistan; ^3^ Division of Science and Technology, Department of Zoology University of Education Lahore Multan Pakistan; ^4^ Department of Animal Science, Faculty of Agriculture, Forestry and Natural Environment Aristotle University of Thessaloniki Thessaloniki Greece; ^5^ Department of Zoology The Islamia University Bahawalpur Bahawalpur Punjab Pakistan; ^6^ Sydney School of Veterinary Science, Faculty of Science The University of Sydney Sydney New South Wales Australia; ^7^ Department of Zoology Bacha Khan University Charsadda Khyber Pakhtunkhwa Pakistan; ^8^ Department of Basic Medical Sciences, College of Medicine AlMaarefa University Riyadh Saudi Arabia; ^9^ Research Center, Deanship of Scientific Research and Post‐Graduate Studies AlMaarefa University Riyadh Saudi Arabia; ^10^ Department of Biology, College of Science King Khalid University Abha Asir Saudi Arabia; ^11^ National Federation of Associations of Medical Practitioners in Chad (FENAPMT) Ministry of Health N'Djamena city Chad; ^12^ Department of Zoology Shaheed Benazir Bhutto University Peshawar Khyber Pakhtunkhwa Pakistan

**Keywords:** *18S rRNA*, epidemiological factors, *Hepatozoon canis*, Pakistani dogs

## Abstract

*Hepatozoon canis* is the causative agent of canine hepatozoonosis that results in lethargy and severe anemia during acute infection in dogs. In this investigation, 654 blood samples were collected from apparently healthy dogs in Punjab and Khyber Pakhtunkhwa (KPK) provinces in Pakistan and screened for the presence of *H. canis* . Phylogenetic diversity of the detected *H. canis* DNA, infection‐associated risk factors and parasite‐induced alterations in the host's complete blood count were also evaluated. PCR‐based screening confirmed the *H. canis* infection in 48 out of 654 (7.3%) dog blood samples. The presence of this apicomplexan protozoa was confirmed through Sanger sequencing. Phylogenetic reconstruction using the *H. canis* *18S rRNA* gene showed that the sequences generated in this study clustered closely with isolates previously reported from India, Australia, Iran, Zambia, China, Spain, and Brazil. The risk factor assessment indicated that parasite prevalence differed significantly between the sampling sites but did not vary between the dog breeds, animal sex, and the presence or lack of ecto‐parasitic infestation in dogs. Reduced hematocrit levels were recorded in *H. canis* infected dogs. In conclusion, *H. canis* was prevalent among the dogs in surveyed regions of Pakistan, with infection rates varying significantly among sampling districts. Furthermore, infected dogs exhibited reduced hematocrit levels, suggesting that *H. canis* infection may contribute to hematological alterations even in apparently healthy animals.

## Graphical abstract

A total of 654 blood samples were collected from healthy dogs in Punjab and Khyber Pakhtunkhwa provinces in Pakistan and screened for the presence of *Hepatozoon canis*. Genetic diversity of the detected *H. canis* DNA, infection‐associated risk factors, and changes in the complete blood count of the host due to parasitic activity were also evaluated. PCR‐based screening revealed that *H. canis* infection was detected in 48 out of 654 (7.3%) dog blood samples. The presence of apicomplexan protozoa was confirmed through Sanger sequencing.

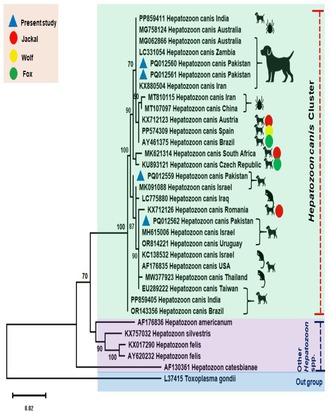

## 1 Introduction

Dogs play an important role in human society worldwide, serving not only as companion animals but also as guard dogs and participants in various sporting activities (Liu et al. 2024). Their close association with humans and frequent exposure to diverse environmental settings increases their risk of contact with arthropod vectors and other disease‐transmitting agents. Consequently, dogs are highly susceptible to a range of vector‐borne infections, many of which can cause significant morbidity and, in severe cases, mortality (Mutlu et al. 2024). Vector‐borne diseases of both pet animals and livestock are a major concern in climatically warm regions like Pakistan, where there is an abundance of vector population (Milošević et al. 2024) due to suitable climatic conditions and insufficient preventive practices (Iatta et al. 2021). Dogs sub‐clinically infected with vector‐borne pathogens serve as reservoirs of these diseases for both their owners and other animals in close contact (Aydin et al. 2025).

*Hepatozoon canis* is a protozoan parasite that belongs to phylum Apicomplexa and placed within the family Hepatozoidae. In addition to domestic dogs, this parasite is known to infect several wild canine species and other carnivores including cat, fox, jackal, raccoon, African wild dog, and hyena (Salim et al. 2019). Diverse hematophagous invertebrate, including fleas, ticks, mites, lice, mosquitoes, sand flies, bugs and tsetse flies are known to be definitive host of *H. canis* and involve in its transmission (Criado‐Fornelio et al. 2018). *Rhipicephalus sanguineus* *sensu lato* are the most common vector of *H. canis* . However, *Ambylomma ovale*, *Dermacentor marginatus* , *Haemaphysalis longicornis* , *H. flava* , and *Rhipicephalus turanicus* are also reported to be suspected vectors of this parasite (Baneth 2011). Signs of this infection in dogs include pyrexia, anorexia, hyperesthesia, muscle wasting and weight loss (Criado‐Fornelio et al. 2018). Infection with *H. canis* has been associated with notable haemato‐biochemical abnormalities in dogs, including anemia, hyperbilirubinemia, lymphocytosis, hyperglobulinemia, hypoalbuminemia and elevated liver aminotransferase activities (Aktas et al. 2013). Currently, no vaccine is available against canine hepatozoonosis and infection control solely depends on vector control (Mutlu et al. 2024). Usually a combination of Imidocarb dipropionate and Doxycycline are used to treat the infected dogs (Mondal et al. 2021).

Regarding the dog population in Pakistan, a recent study reports that there are 190,200 registered dogs and over 3 million stray dogs in the country (Liu et al. 2024). *H. canis* has not been screened frequently in Pakistani dogs. There are few documented reports regarding the presence of this parasite in local dogs and most of these reports are from the Punjab province (Qamar et al. 2017; Ahmad et al. 2018). This limited available data in literature urged us to conduct this molecular survey in five districts that are situitated in two provinces of Pakistan. This investigation aimed to report the prevalence and the phylogeny of *H. canis* among dogs in Muzaffargarh, Bahawalpur, Jhang, Dir Upper and Charsadda including a note on its impact on the hematological profile of infected dogs.

## 2 Materials and Methods

### 2.1 Study Areas

**FIGURE 1:**
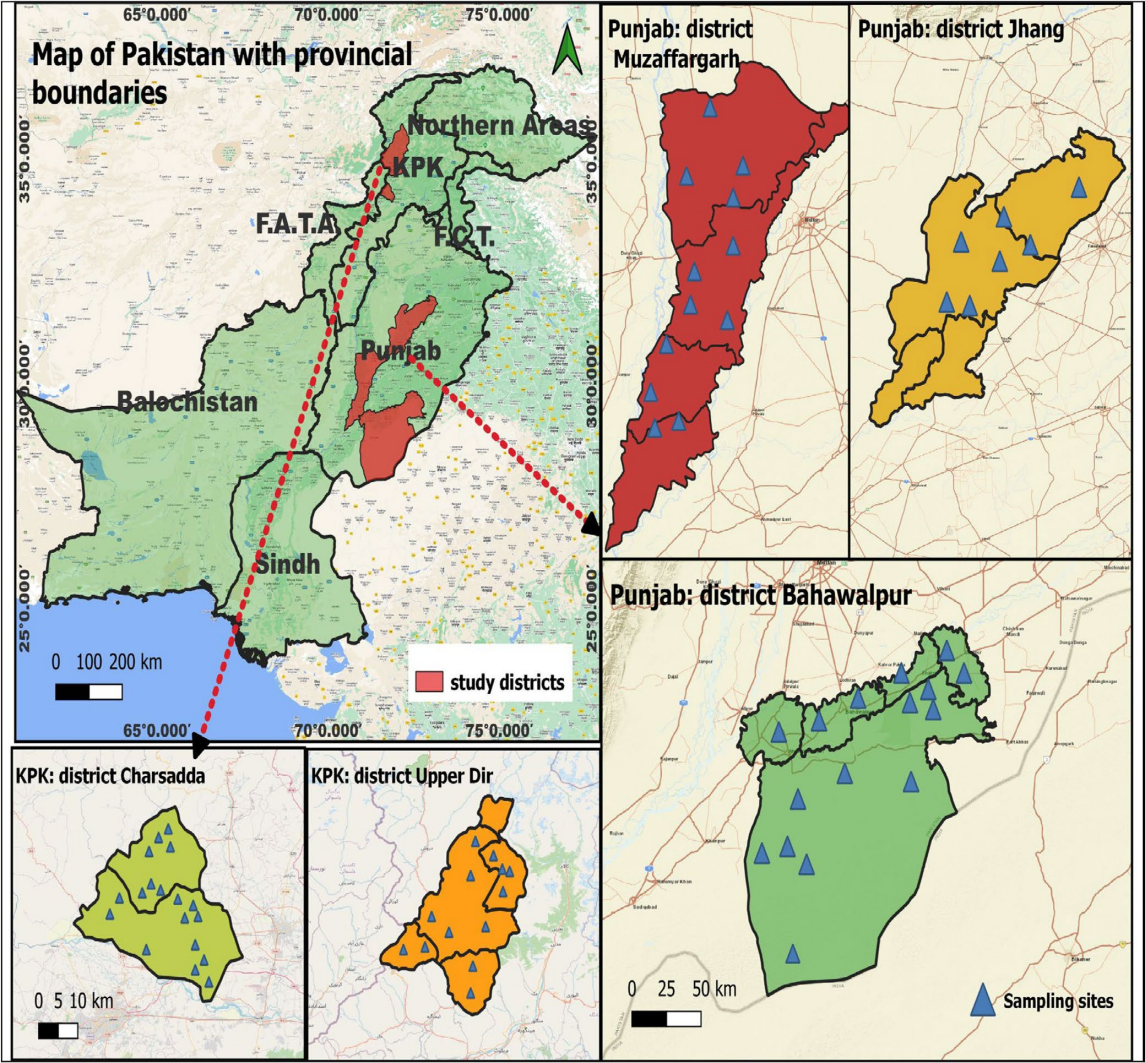
Map of Pakistan showing Punjab and Khyber Pakhtunkhwa provinces. Magnifications are presenting the sampling sites in both provinces. The map images were created with QGIS software and shape files downloaded from DIVA‐GIS that were listed as Free Spatial Data.

Sample collection was conducted in three districts of Punjab Jhang, Muzaffargarh, and Bahawalpur and two districts of Khyber Pakhtunkhwa (KPK): Upper Dir and Charsadda. All these sampling sites were geographically different, and we hypothesized that we may observe variation in *H. canis* infection rate between these districts (Figure 1).

### 2.2 Experimental Design

We used Solvin's formula to estimate sample size during the present study. Solvin's formula was computed as follows: *n* = N / (1 + *N* × e2). Whereas: *n* = no. of samples, *N* = total dog population obtained from Pakistan Bureau of Statistics (https://www.pbs.gov.pk/agriculture‐census/), *e* = margin of error (Thrusfield 1995). A total of 654 dog blood samples were collected following informed oral consent from dog owners during February 2023 to May 2024. All the dogs were adult and apparently healthy. Puppies and sick dogs were not included in this study. The majority of the blood samples were collected from Punjab (*N* = 475). Fifty different dog breeds were recognized among the enrolled dogs and samples were collected at home or at the agricultural lands following the invitation of the dog owners. The district wise sample collection was as follows: Muzaffargarh (*N* = 256), Bahawalpur (*N* = 123), and Jhang district (*N* = 96). From Khyber Pakhtunkhwa (KPK), a total of 179 dogs were enrolled, including 134 from Upper Dir and 45 from Charsadda. Each animal's' body was thoroughly examined to record the presence of ectoparasites (mainly ticks). Data was gathered through a screening sheet by interviewing the dog owners at the sample collection site to estimate the risk factors that were potentially associated with the infection rate of *H. canis* .

### 2.3 Blood Sample Collection and Complete Blood Counting

Two to three milliliter blood was aseptically collected from each enrolled dog from the saphenous vein in the limb of most dogs, especially the watch dogs. Blood samples were collected from the jugular vein of a few dogs and preserved in labeled EDTA‐coated tubes and analyzed by using a hematology analyzer (Sysmex KX21, Hyogo, Japan). Complete blood count parameters including white blood cell count, granulocytes (count and %), lymphocytes (count and %), red blood cell count, hemoglobin, hematocrit, mean corpuscular hemoglobin, mean cell volume, mean corpuscular hemoglobin concentration, red cell distribution width‐standard deviation with coefficient of variation (%) and red cell distribution width‐standard deviation were determined for enrolled animals.

### 2.4 DNA Extraction and Molecular Detection of *Hepatozoon canis* by PCR

Whole blood of each dog was targeted for DNA extracted by using Genomic DNA extraction Kit (Promega, Madison, WI, USA). Gel electrophoresis was performed for DNA confirmation prior to its use in PCR. The primer pair (5′ GTT TCT GAC CTA TCA GCT TTC GAC G 3′ and 5′ C AAA TCT AAG AAT TTC ACC TCT GAC 3′) targeted the *18S rRNA* gene of *H. canis* (600 bp) following Ujvari et al. (2004). A 50 μL reaction mixture was prepared containing template DNA (50 ng), 1X PCR buffer, dNTP (0.125 mM of each), MgCl_2_ (1.5 mM), primers (0.6 μM each), and 1 U of *Taq* DNA polymerase (AbClonal USA). PCR amplification was carried out by using a DNA thermal cycler (Gene Amp PCR system 2700, Applied Biosystems, UK). The thermo‐profile constituted an initial denaturing step at 94°C for 5 min followed by 35 cycles of denaturing step at 94°C for 30 s, an annealing step at 61°C for 30 s, and an extension step at 72°C for 30 s. This was followed by a final extension at 72°C for 5 min (Ujvari et al. 2004). During each reaction, distilled water and DNA of *Hepatozoon* sp. (amplified from rodent blood during our previous study) were used as negative and positive controls, respectively. PCR‐amplified products were resolved by submerged gel electrophoresis employing 1.8% agarose gel, and a UV trans‐illuminator was used for the visualization of amplicon, and results were recorded through a digital camera.

### 2.5 DNA Sequencing and Genetic Diversity of *Hepatozoon canis*

From the *18S rRNA* gene amplicons of *H. canis* that were detected during present study, we randomly selected 10 for confirmation by DNA sequencing: Two samples were selected each from Muzaffargarh, Bahawalpur and Jhang district in Punjab and from Dir Upper and Charsadda in KPK. DNA sequencing facility was provided by a commercial lab (First Base, Malaysia). As they all represented the same parasite, we had randomly included 4 sequences for the phylogenetic analysis. The generated sequences were initially trimmed through FinchTV (version 1.4.0) to remove the misread nucleotides as well as the primer binding sites. The sequences were uploaded to NCBI GenBank, where they were assigned unique accession numbers PQ012559–PQ012562. BLAST analysis was performed to obtain other similar *18S rRNA* sequences and all of them were aligned by using ClustalW in BioEdit (version 7.2.5, https://bioedit.software.informer.com/7.2/) and then they were imported into MEGA X (version 10.2.6, https://www.megasoftware.net/). The best‐fit model was selected for phylogenetic tree creation based on lowest Akaike Information Criteria (AIC) and Bayesian Information Criteria (BIC) values with 1000 bootstraps. Final tree was inferred by using iTOL server (https://itol.embl.de/). *18S rRNA* gene of *Hepatozoon americanum* (AF176836), *Hepatozoon silvestris* (KX757032), *Hepatozoon felis* (KX017290 and AY620232), *Hepatozoon catesbianae* (AF130361) and *Toxoplasma gondii* (L37415) were used as an out group during the phylogenetic analysis.

### 2.6 Statistical Analysis

Data analysis was done by using Minitab (version 18, Chicago, USA, https://www.minitab.com/). One way ANOVA was applied to compare *H. canis* prevalence among dog breeds. Comparison of parasite prevalence between the sampling sites was done through Chi‐squared test. Correlation between the studied risk factors and the presence of *H. canis* was analyzed through Fisher's exact test. Two sample *t*‐test was conducted to compare CBC parameters between *H. canis* positive and negative dogs. *p* ≤ 0.05 was considered statistically significant.

## 3 Results

### 3.1 Molecular Prevalence of *Hepatozoon canis*

A 600 base pair fragment from the *18S rRNA* gene of *H. canis* was amplified in 48 out of 654 (7%) dog blood samples that were screened in this study (Table 1).

**TABLE 1:** *Hepatozoon canis* prevalence among dogs enrolled from five districts of Pakistan.

Province	Districts	*N*	*Hepatozoon canis* positive	*Hepatozoon canis* negative	Chi square value	*p*
Punjab	Muzaffargarh	256	29/256 (11%)	227/256 (89%)		
	Jhang	96	4/96 (4%)	92/96 (96%)		
	Bahawalpur	123	7/123 (6%)	116/123 (94%)		
Khyber Pakhtunkhwa	Charsadda	45	2/45 (4%)	43/45 (96%)	10.07	0.04*
	Upper Dir	134	6/134 (4%)	128/134 (96%)		
	Grand Total	654	48/654 (7%)	606/654 (93%)		

*Note:* *N* represents the total number of samples collected from each district. % prevalence is given in parentheses. *p*‐value represents the output of Chi‐square test. *p* < 0.05 = Least significant (*).

### 3.2 Phylogenetic Analysis of *Hepatozoon canis* Based on *18S rRNA* Gene

**FIGURE 2:**
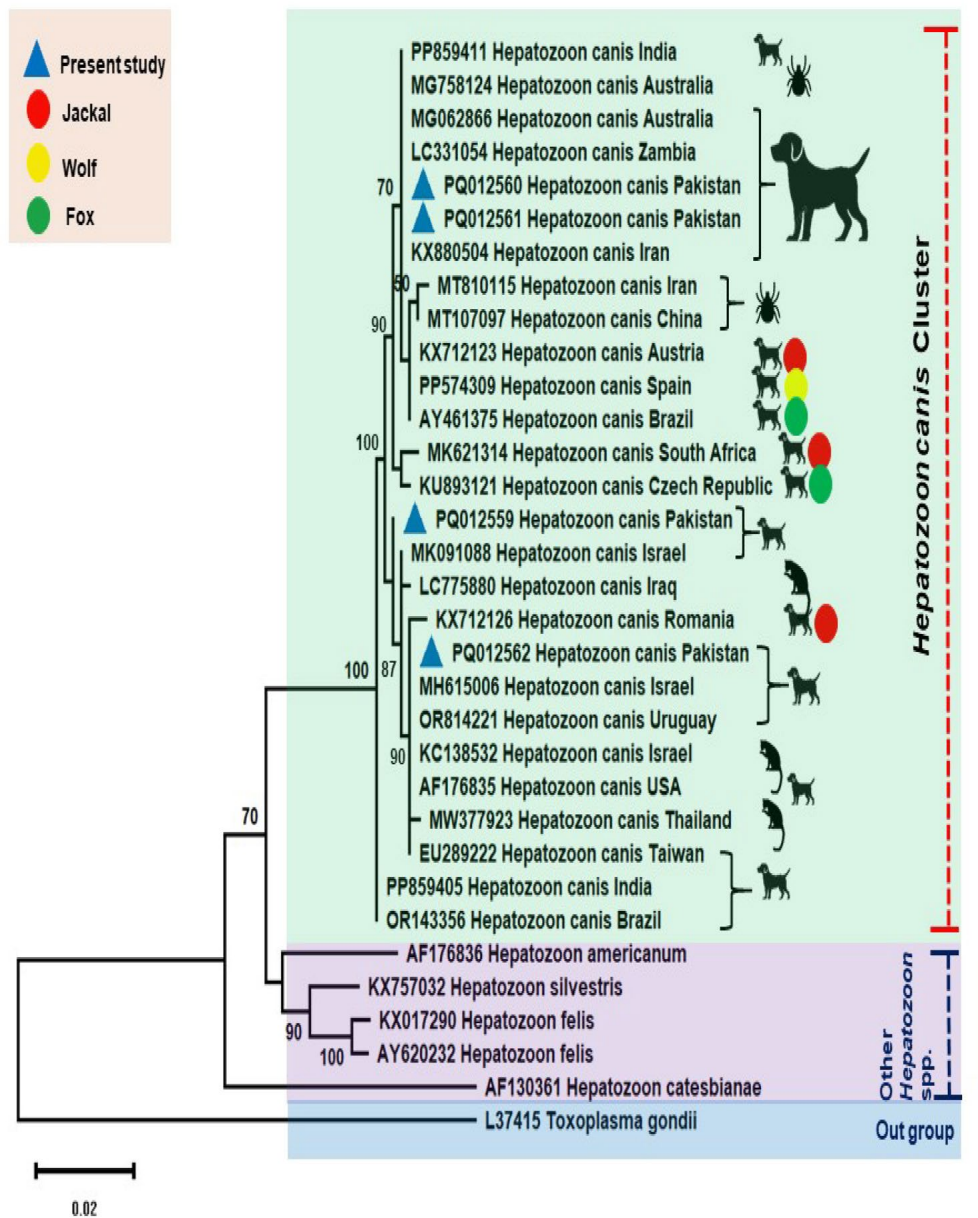
Maximum likelihood tree generated by MEGA X software based on Kimura‐2 parameter model was used for the multiple alignments of partial *18S rRNA* sequences (600 bp) from *Hepatozoon canis* isolated in this study and those available in GenBank from other countries around the world. The four new *18S rRNA* sequences of *H. canis* obtained are marked with a triangle. The scale bar represents 0.02 substitutions per nucleotide position. Bootstrap value is shown as a number on each node. Vector images used in this figure were downloaded from a free website (http://www.freepik.com).

Partial *18S rRNA* gene sequence analysis for *H. canis* during phylogenetic analysis revealed that the amplified nucleotides generated during this investigation were genetically distinct from each other as they resided in three different clusters as shown in Figure 2. Amplified haplotypes PQ012560 and PQ012561 showed genetic similarity, and they were grouped with the *H. canis* sequence reported from India (PP859411), Australia (MG758124 and MG062866), Zambia (LC331054), Iran (KX880504 and MT810115), China (MT107097), and Austria (KX712123). The third Pakistani haplotype (PQ012559) clustered with *18S rRNA* sequences of *H. canis* deposited from Spain (PP574309), Brazil (AY461375), South Africa (MK621314), Czech Republic (KU893121), Israel (MK098081), and Iraq (LC775880). While the fourth Pakistani haplotype (PQ012562) clustered with *18S rRNA* sequences of *H. canis* deposited in GenBank from Romania (KX712126), Israel (MH615006 and KC138532), Uruguay (OR814221), USA (AF176835), Thailand (MW377923) Taiwan (EU289222), India (PP859405), and Brazil (OR143356) (Figure 2).

### 3.3 Risk Factor Analysis

During the present investigation, 8.4% of dogs from Punjab and 4.5% of dogs from KPK were *H. canis* infected (Table 1). Dogs from all sampling districts had *H. canis* infection. Results of the Chi‐squared test indicated that the prevalence of *H. canis* varied significantly among the dogs enrolled from five Pakistani districts (*p* = 0.04). Dogs from Muzaffargarh had the highest infection rate (11%), followed by Bahawalpur (6%), Charsadda (4%), Dir Upper (4%), and Jhang (4%) (Table 1).

We found that 20/55 of the enrolled dog breeds were infected with *H. canis* . Results of one‐way ANOVA revealed that *H. canis* infection was not limited to a specific dog breed during the current investigation (*p* = 0.8) (Supplementary Table S1).

Data analysis of risk factors indicated that *H. canis* infection was not limited to a particular dog sex (*p* = 0.5) or to the ectoparasites infesting the enrolled dogs (*p* = 0.2) (Table 2).

**TABLE 2:** Association of the studied risk factors with the prevalence of *Hepatozoon canis* among the dogs enrolled from five sampling districts during the present study.

Parameters		*Hepatozoon canis* positive	*Hepatozoon canis* negative	*p*
Gender	Male	36/456 (08%)	420/456 (92%)	0.5
	Female	12/198 (06%)	186/198 (94%)	
Ectoparasites	Yes	22/244 (09%)	222/244 (91%)	0.2
	No	26/410 (06%)	384/410 (94%)	

*Note:* Prevalence of pathogen (%) is given in parenthesis. *p*‐value represents the results of Fischer's exact test calculated for each studied parameter. *p* > 0.05 = Non significant.

### 3.4 Complete Blood Count Analysis

Hematocrit (%) was the only parameter among the screened CBC parameters that was significantly reduced (*p* = 0.05) among the *H. canis‐*infected dogs when compared to uninfected dogs (Table 3).

**TABLE 3:** Comparison of studied complete blood count parameters between *Hepatozoon canis* infected and uninfected dogs enrolled from five districts during the present study.

Parameters	*Hepatozoon canis* +ve	*Hepatozoon canis* −ve	*p*
White blood cell (×10^9^/L)	13.20 ± 2.3	13.10 ± 0.4	01
Lymphocytes (×10^9^/L)	8.96 ± 1.9	8.95 ± 0.34	01
Granulocytes (×10^9^/L)	2.11 ± 0.64	2.89 ± 0.22	0.3
Lymphocytes (%)	67.1 ± 7	68.8 ± 1.5	0.8
Granulocytes (%)	18.4 ± 4.3	20.9 ± 1.3	0.6
Red blood cells (×10^12^/L)	4.33 ± 0.6	5.31 ± 0.14	0.2
Hemoglobin (gd/L)	11.13 ± 0.8	12.5 ± 0.2	0.1
Hematocrit (%)	26.3 ± 4.4	35.6 ± 1.0	0.05*
Mean cell volume (fL)	58.6 ± 3.8	64.6 ± 0.8	0.1
Mean corpuscular hemoglobin (pg)	40.9 ± 14	31.1 ± 3.2	0.5
Mean corpuscular hemoglobin concentration (g/dL)	78 ± 39	54 ± 7.6	0.6
Red cell distribution width‐standard deviation (fL)	17.9 ± 3.3	22.2 ± 0.9	0.2
Red cell distribution width‐coefficient of variation (%)	20.5 ± 4.3	27.3 ± 1.0	0.1

*Note:* Data is presented as mean + standard error of the mean.

* Represents the significant output of two sample test calculated for the studied parameters. *p* > 0.05 = Non significant; *p* < 0.05 = Least significant (*).

## 4 Discussion

Dogs worldwide have been reported to harbor tick‐borne parasites that results in their significant morbidity and mortality (Ahmad et al. 2025; Dumanli et al. 2025; Chen et al. 2025). The present investigation revealed an overall prevalence of 7.3% *H. canis* DNA in the enrolled dogs from the two provinces in Pakistan. There are three previous reports from Pakistan in which the prevalence of *H. canis* has been reported among local dogs. In a recent study that was conducted in Iran and Pakistan simultaneously, Iatta et al. (2021) found that 63% (Infected/Total dog number) of the dogs enrolled from Bahawalpur in Punjab were infected with *H. canis* . Ahmad et al. (2018) collected 341 dog blood samples from Kasur, Rawalpindi and Muzaffargarh districts in Punjab and found that 45.5% (Infected/Total dog number) of the screened dogs were infected with *H. canis* . Qamar et al. (2017) found that 18 out of 151 dog blood samples (11.9%) that were collected from Lahore, Islamabad and Multan districts in Punjab were infected with *H. canis* . *Hepatozoon* canis infection rate observed during present investigation is lower than the previous studies from Pakistan. There are reports of *H. canis* infection in dog from various countries across the globe: 78.4.1% in Iran (Iatta et al. 2021), 63% in Mexico (Jarquín‐Díaz et al. 2016), 58.7% in Brazil (Spolidorio et al. 2009), 47.5% in Cuba (Díaz‐Sánchez et al. 2021), 26% in Hungary (Hornok et al. 2013), 22.3% in Turkey (Aktas et al. 2015), 19.3% in Poland (Tołkacz et al. 2023), 14% and 18% in Italy (Pacifico et al. 2020; Chisu et al. 2023), 11.4% in Thailand (Jittapalapong et al. 2006), 5.5% in Palestine (Azmi et al. 2017), 4.5% in Colombia (Ramírez‐Alvarado et al. 2024) and 2.04% in China (Guo et al. 2020). The variations in *H. canis* prevalence might be due to different climatic and geographical conditions, sampling season, age, breed, the gene amplified, host immunity, and the tick density in the study areas (Qamar et al. 2017).

For the molecular analysis of the evolutionary history of vertebrates, *18S rRNA* genes are among the most commonly used genes due to their slow evolutionary rates (Wu et al. 2015). The phylogenetic tree constructed using the *18S rRNA* nucleotide sequences of *H. canis* obtained from dogs in this study along with those retrieved from GenBank were highly identical with *H. canis* sequences of dogs, wolves, jackals, foxes, and ectoparasites from different parts of the world (Guo et al. 2020; Greay et al. 2018; Qiu et al. 2018; Mitková et al. 2017, 2016; Criado‐Fornelio et al. 2006; Fernandes et al. 2024; Viljoen et al. 2020; Léveillé et al. 2019; Baneth et al. 2013; Mathew et al. 2000). This limited information regarding the genetic diversity of *H. canis* available in literature demands that the prevalence of *H. canis* should be determined in other canines along with the dogs from various areas having different geography and climatic conditions. Vectors infesting the dogs must also be screened to understand their details of vector‐parasite interactions as they are required for the effective control of this common protozoan parasite.

Risk factor analysis revealed that *H. canis* prevalence among dogs varied significantly among the dogs screened at various sampling sites in this study. We observed a higher infection rate of *H. canis* in dogs enrolled from various areas in Punjab as compared to those screened in KPK (Table 1). This variation in *H. canis* prevalence among sampling districts may reflect differences in environmental conditions, vector abundance, and host exposure to infected ticks. The sampling sites in Punjab are mostly arid plain areas with higher humidity and temperatures that are conducive for tick growth and reproduction as compared to the sampling sites in KPK, where the temperatures are moderate to cold as compared to Punjab, limiting the tick growth and disease transmission. The higher infection rate observed in Muzaffargarh could be associated with its warm climate and agricultural landscape, which may favor tick survival and increase opportunities for host‐vector contact (Ahmad et al. 2018). In addition, variations in dog management practices, tick‐control measures, and access to veterinary care among districts may have contributed to the observed differences (Iatta et al. 2021). However, further studies incorporating vector surveillance and environmental risk factor analyses are needed to confirm the factors underlying these geographical variations. A similar trend was reported by Qamar et al. (2017) as they had also reported that *H. canis* infection varied significantly among the dogs that were enrolled from Islamabad, Lahore, and Multan cities in Punjab, Pakistan. A similar trend was also reported by Ahmad et al. (2018) among dogs enrolled from Punjab in Pakistan. Ramírez‐Alvarado et al. (2024) had also documented that dogs enrolled from the rural areas of Colombia had a higher *H. canis* infection rate than those enrolled from the urban areas. Iatta et al. (2021) had also found that dogs enrolled from Iran had a significantly higher rate of *H. canis* infection than those enrolled in their study from Pakistan.

The absence of a significant association between *Hepatozoon canis* infection and dog breed, sex, or ectoparasite infestation suggests that exposure to the parasite may occur relatively uniformly across canine populations in the study areas. Because *H. canis* infection is primarily acquired through ingestion of infected ticks rather than through tick attachment alone, the presence of ectoparasites at the time of sampling may not accurately reflect previous exposure to infected vectors (Jarquín‐Díaz et al. 2016). Similarly, neither breed nor sex is expected to confer substantial biological resistance or susceptibility to infection, particularly in regions where dogs are exposed to similar environmental conditions and vector populations (Qamar et al. 2017). The lack of significant associations may also indicate that ecological and management‐related factors, such as local vector abundance, housing conditions, roaming behavior, and tick‐control practices, play a greater role in determining infection risk than host demographic characteristics (Hornok et al. 2013). In addition, the relatively low prevalence of infection may have limited the statistical power to detect subtle differences among groups. Similar to our observations, Qamar et al. (2017) also did not find any association between the dog breeds or sex, age, or presence of ecto‐parasites on dog with the presence of *H. canis* among Pakistani dogs. Similar results have been reported from Iran and Hungary (Iatta et al. 2021; Hornok et al. 2013). Contrary to these observations, higher *H. canis* infection had been associated with age, sex, and hair coat length in dogs from Italy, Türkiye, and Colombia (Aktas et al. 2015; Chisu et al. 2023; Ramírez‐Alvarado et al. 2024).

Complete blood count analysis is a routine blood test that can be used to determine the health status of dogs (Ali et al. 2024; Liu et al. 2026). It has been established that sporozoites of *H. canis* invade the mononuclear cells and are subsequently disseminated via the bloodstream or lymphatic system to multiple target organs associated with lymphoid tissues (bone marrow, spleen, and lymph nodes) as well as to other internal organs like kidney, liver and lungs (Otranto et al. 2011). The reduced hematocrit observed in *H. canis* ‐infected dogs during this investigation suggests that infection may adversely affect erythrocyte homeostasis and contribute to the development of mild anemia. Although red blood cell count, hemoglobin concentration and mean cell volume did not differ significantly between infected and uninfected dogs, their tendency to be lower in infected animals may indicate early or subclinical hematological disturbances. The absence of significant differences in these parameters could be related to low parasitemia levels and variation in the stage of infection (Chimedtseren et al. 2025). Similar hematological alterations have been reported in canine hepatozoonosis and are thought to result from chronic inflammation, increased erythrocyte destruction, or suppression of erythropoiesis associated with parasitic infection (Chhabra et al. 2013). Díaz‐Sánchez et al. (2021) had reported that *H. canis* infected dogs in Cuba were presenting the signs of anemia and leukocytosis (Díaz‐Sánchez et al. 2021). The observations we made during present investigation are in close agreement with Qamar et al. (2017) as they had documented that *H. canis* infected dogs from Pakistan exhibited higher relative distribution width of red blood cells, elevated minimum inhibitory dilution with lower hematocrit and mean hemoglobin content than the dogs in which *H. canis* infection was not detected. Similar to our observations, low mean erythrocyte, hemoglobin and hematocrit values and high leukocyte count were reported in *H. canis* infected dogs from India (Chhabra et al. 2013) as well as hematological abnormalities and eosinophilia in dogs from Italy (Otranto et al. 2011). Contrary to these observations, Díaz‐Sánchez et al. (2021) compared the hematological parameters between *H. canis* infected and non‐infected dogs and found no significant differences among them. As we have only screened *H. canis* among the enrolled dogs, it would be worth mentioning that the hematological changes observed during this investigation could be due to the presence of other blood borne pathogens that remained not investigated.

The present study has certain limitations that should be considered when interpreting the findings. Sampling was restricted to selected districts of Pakistan, and therefore the results may not fully represent the epidemiological situation of *H. canis* across the country. The sample size in the present study was estimated using Slovin's formula based on dog population estimates obtained from the Pakistan Bureau of Statistics. Although this approach facilitated sampling across a broad geographic area, it does not incorporate epidemiological parameters such as expected prevalence and confidence intervals. Therefore, the sampled dogs may not fully represent the demographic and epidemiological diversity of the canine population in the surveyed regions, and the prevalence estimates should be interpreted accordingly. Another limitation of this study is that only a subset of the PCR‐positive samples was subjected to sequencing and phylogenetic analysis. Consequently, the reported haplotype diversity may not fully capture the genetic variation of *H. canis* circulating within all sampled regions. Future studies involving larger sequencing datasets and additional genetic markers are needed to provide a more comprehensive assessment of parasite diversity. In addition, environmental and vector‐related factors that may influence transmission dynamics were not evaluated. Consequently, the identified prevalence patterns should be interpreted within the context of the sampled populations. Future studies involving larger sample sizes, broader geographical coverage, and integrated vector surveillance are warranted to provide a more comprehensive understanding of the epidemiology of canine hepatozoonosis in Pakistan.

## 5 Conclusion

We are reporting the prevalence of *H. canis* among dog populations from two provinces in Pakistan confirming the endemic nature of this parasite. Prevalence of *H. canis* varied between the dogs enrolled from different sampling sites but this infection was not associated with a specific dog breed or with their sex or age group. Significantly decreased hematocrit levels were observed in *H. canis* infected dogs. The findings of this study enhance current understanding of *H. canis* in dogs from Pakistan and will aid in the planning and implementation of effective control measures for canine vector‐borne diseases.

## Author Contributions

**Madiha Rasool:** conceptualization (equal), writing – original draft (equal). **Muhammad Latif:** data curation (equal), methodology (equal). **Ioannis A. Giantsisis:** resources (equal), writing – review and editing (equal). **Muhammad Ali:** formal analysis (equal), writing – original draft (equal). **Hira Muqaddas:** data curation (equal), writing – original draft (equal). **Muhammad Naeem:** software (equal), writing – original draft (equal). **Sami Ul Haq:** writing – original draft (equal), writing – review and editing (equal). **Gehan M. Elossaily:** data curation (equal), investigation (equal). **Esmael M. Alyami:** supervision (equal), writing – review and editing (equal). **Mona Alsolami:** investigation (equal), supervision (equal). **Ousman Brahim Mahamat:** writing – original draft (equal), writing – review and editing (equal). **Irfan Ullah:** validation (equal), writing – original draft (equal). **Asmat Ullah Khan:** data curation (equal), methodology (equal). **Adil Khan:** software (equal), writing – original draft (equal). **Furhan Iqbal:** conceptualization (equal), writing – original draft (equal), writing – review and editing (equal).

## Funding

This work is supported by King Khalid University, through the Large Research Project under grant number RGP1/89/46, and AlMaarefa University through research project number MHIRSP2025‐018.

## Ethics Statement

All the animal handling procedures and experimental procedures described in this study were reviewed and granted approval by the Ethics Committee of Bahauddin Zakariya University, Multan, Pakistan. All animal experimentation was conducted in accordance with applicable laws, regulations, and guidelines, prioritizing animal welfare and minimizing any potential harm. The experimentation was conducted in accordance with applicable laws, and ARRIVE guidelines.

## Consent

The authors have nothing to report.

## Conflicts of Interest

The authors declare no conflicts of interest.

## Supporting information


**Table S1:** Prevalence of *Hepatozoon canis* among the various dog breeds enrolled during the present study from five sampling districts. % prevalence of pathogen is given in parentheses. The *p*‐value represents the results of the One‐way ANOVA test calculated for each sampling site.

## Data Availability

The datasets generated and/or analyzed during the current study are available in the GenBank repository with accession numbers PQ012559, PQ012560, PQ012561, and PQ012562 (https://www.ncbi.nlm.nih.gov/nuccore/PQ012559; https://www.ncbi.nlm.nih.gov/nuccore/PQ012560; https://www.ncbi.nlm.nih.gov/nuccore/PQ012561; https://www.ncbi.nlm.nih.gov/nuccore/PQ012562).
