# Supplementary material for: Molecular Genetics of Hepatozoon canis With a Note on Effect of Parasite on the Complete Blood Count Profile of Canine Hosts
Source: Ecol Evol. 2026 Aug 5;16(8):e74039. doi: 10.1002/ece3.74039 (PMC13439630; doi:10.1002/ece3.74039)
Supplement: Supplementary file 1 [file ECE3-16-e74039-s001.docx]

**Supplementary Table 1.** Prevalence of *Hepatozoon canis* among the various dog breeds enrolled during the present study from five sampling districts. % prevalence of pathogen is given in parentheses. The P-value represents the results of the One-way ANOVA test calculated for each sampling site.

| **Dog breeds** | **N** | ***Hepatozoon canis* positive** | ***Hepatozoon canis* negative** | ***P*-value** |
| --- | --- | --- | --- | --- |
| Stray | 162 | 12/162 (7%) | 150/162 (93%) |  |
| Local | 63 | 2/63 (3%) | 54/63 (97%) |  |
| Kathia | 54 | 8/54 (15%) | 46/54 (85%) |  |
| German Shepherd | 36 | 1/36 (3%) | 35/36 (97%) |  |
| Alsheehan | 33 | 3/33 (9%) | 30/33 (91%) |  |
| Bull Terrier | 28 | 5/28 (18%) | 23/28 (82%) |  |
| Boli | 25 | 0/25 (0%) | 25/25 (100%) |  |
| German | 24 | 1/24 (4%) | 23/24 (96%) |  |
| Barfani | 19 | 1/19 (5%) | 18/19 (95%) |  |
| Pointer | 18 | 3/18 (17%) | 15/18 (83%) |  |
| Russian | 15 | 1/15 (7%) | 14/15 (93%) |  |
| Cross Hound | 15 | 0/15 (0%) | 15/15 (100%) |  |
| Gultair | 19 | 1/19 (5%) | 18/19 (95%) |  |
| Bhaggyari | 13 | 0/13 (0%) | 13/13 (100%) |  |
| Tazy | 12 | 0/12 (0%) | 12/12 (100%) |  |
| Poodle | 10 | 2/10 (20%) | 8/10 (80%) |  |
| Common | 10 | 1/10 (10%) | 9/10 (90%) |  |
| Aspin | 10 | 0/10 (0%) | 10/10 (100%) |  |
| Shikari | 10 | 0/10 (0%) | 10/10 (100%) | 0.8 |
| Pista | 08 | 0/08 (0%) | 8/8 (100%) |  |
| Labrador | 07 | 2/07 (29%) | 5/7 (71%) |  |
| Bully | 07 | 1/7 (14%) | 6/7 (86%) |  |
| Grey Hound | 06 | 0/6 (0%) | 6/6 (100%) |  |
| Cross | 04 | 1/4 (25%) | 3/4 (75%) |  |
| English | 04 | 1/4 (25%) | 3/4 (75%) |  |
| Siberian Husky | 04 | 0/4 (0%) | 4/4 (100%) |  |
| Moti | 03 | 0/3 (0%) | 3/3 (100%) |  |
| Beacho | 03 | 0/3 (0%) | 3/3 (100%) |  |
| Belgium | 03 | 0/3 (0%) | 3/3 (100%) |  |
| Rottweiler | 03 | 1/3 (33%) | 2/3 (67%) |  |
| Tona | 02 | 0/2 (0%) | 2/2 (100%) |  |
| Pit Bull | 02 | 1/2 (50%) | 1/2 (50%) |  |
| Afghani | 02 | 0/2 (0%) | 2/2 (100%) |  |
| Kokoni | 02 | 0/2 (0%) | 2/2 (100%) |  |
| Persian | 02 | 0/2 (0%) | 2/2 (100%) |  |
| Lavery | 02 | 0/2 (0%) | 2/2 (100%) |  |
| Lab Cross | 02 | 0/2 (0%) | 2/2 (100%) |  |
| Retriever | 02 | 0/2 (0%) | 1/2 (100%) |  |
|  |  |  |  |  |
| Dobermann | 01 | 0/1 (0%) | 1/1 (100%) |  |
| Jack Russel | 01 | 0/1 (0%) | 1/1 (100%) |  |
| Great Pyrenees | 01 | 0/1 (0%) | 1/1 (100%) |  |
| Akbash | 01 | 0/1 (0%) | 1/1 (100%) |  |
| Kombai | 01 | 0/1 (0%) | 1/1 (100%) |  |
| Argentina | 01 | 0/1 (0%) | 1/1 (100%) |  |
|  |  |  |  |  |
| Gulfari | 01 | 0/01 (0%) | 1/1 (100%) |  |
| Jay Kay | 01 | 0/01 (0%) | 1/1 (100%) |  |
| Bolt | 01 | 0/01 (0%) | 1/1 (100%) |  |
| Pig | 01 | 0/01 (0%) | 1/1 (100%) |  |
|  |  |  |  |  |
|  |  |  |  |  |

*P* > 0.05 = Non significant
